# Influence of Concave Versus Convex Emergence Profiles on Midfacial Mucosal Stability—A Systematic Review With Meta‐Analysis

**DOI:** 10.1111/jerd.70210

**Published:** 2026-06-20

**Authors:** Miha Pirc, Priscilla Balzarini, Jonathan Esquivel, Anina N. Zuercher, Ronald E. Jung, Franz J. Strauss

**Affiliations:** ^1^ Clinic of Reconstructive Dentistry, Center for Dental Medicine University of Zurich Zurich Switzerland; ^2^ Faculty of Medicine, University of Ljubljana Ljubljana Slovenia; ^3^ Private Practice Zurich Switzerland; ^4^ Private Practice Prosthodontist, Metairie Louisiana USA; ^5^ Universidad Autonoma de Chile Santiago Chile; ^6^ Discipline of Periodontics School of Dentistry, Faculty of Medicine and Health, The University of Sydney Sydney New South Wales Australia

**Keywords:** dental abutments, dental implants, dental prosthesis, gingival recession, implant‐supported, prosthodontics

## Abstract

**Objectives:**

To systematically evaluate the influence of concave versus convex emergence profiles on midfacial mucosal stability in partially edentulous patients restored with implant‐supported single restorations.

**Materials and Methods:**

A systematic review and meta‐analysis of randomized controlled trials (RCTs) was conducted and registered in PROSPERO (CRD420251139042). An electronic search in MEDLINE (PubMed) and Embase was performed up to May 7, 2026. Eligible studies included RCTs comparing concave (test group) and convex (control group) transmucosal prosthetic designs and reporting midfacial mucosal level changes (mm). Data extraction and risk of bias assessment (RoB 2) were performed independently. A random‐effects meta‐analysis was conducted using weighted mean differences (MDs) and 95% confidence intervals (CIs). Heterogeneity was assessed using the I^2^ statistic and sensitivity analyses were performed to evaluate the robustness of the findings.

**Results:**

Four RCTs including 144 implants with a 12‐month follow‐up were included. Of these, 128 implants contributed to the quantitative synthesis, with 66 implants allocated to the concave/modified emergence profile group and 62 implants allocated to the convex/non‐concave emergence profile group. The pooled analysis demonstrated a mean difference of −0.31 mm (95% CI −0.63 to 0.02; *p* = 0.064), indicating a trend toward greater midfacial mucosal recession with convex emergence profiles compared with concave designs. Between‐study heterogeneity was low to moderate (*I* ^2^ = 28%), suggesting consistent findings across studies. Sensitivity analyses confirmed the direction of the effect, with pooled estimates ranging from −0.12 to −0.43 mm. Exclusion of one study resulted in a statistically significant difference favoring concave emergence profiles (−0.43 mm; 95% CI −0.70 to −0.16; *p* = 0.002).

**Conclusions:**

Convex emergence profiles are associated with a tendency toward increased midfacial mucosal recession. Concave profiles are preferable to support peri‐implant soft‐tissue stability.

**Clinical Relevance:**

Emergence profile design should be considered an integral component of prosthetic and surgical planning. The use of concave transmucosal contours during provisionalization and definitive restoration may contribute to improved peri‐implant soft tissue stability and enhanced esthetic outcomes.

## 1 Introduction

Implant therapy has evolved from a predominantly bone‐oriented intervention to a prosthetically driven treatment concept, in which the requirements of the definitive restoration guide implant placement. Modern implant planning emphasizes precise three‐dimensional positioning—apico‐coronal, mesio‐distal and bucco‐oral—as well as a proper axial inclination of the implant to enable restorative designs that align with biological and esthetic principles [1, 2, 3, 4, 5]. Inadequate implant positioning may compromise restorative contours, restrict material selection, limit prosthetic options, affect esthetics and negatively affect peri‐implant tissue stability [6, 7, 8].

From a clinical perspective, stable peri‐implant soft tissues are essential, as they directly influence the esthetic outcome of implant‐supported restorations. In contrast, soft‐tissue instability may lead to esthetic discrepancies, patient dissatisfaction, and the need for additional interventions, thereby increasing treatment morbidity and complexity [9, 10, 11, 12]. For this reason, implant‐supported restorations should be carefully evaluated before and during the provisionalization phase, allowing the final restoration to replicate an optimized and biologically compatible restorative design [2, 13].

Within this restorative framework, particular attention must be paid to the transmucosal region connecting the implant and implant‐supported restoration, commonly referred to as the emergence profile (EP). The EP describes the contour of the restoration as it transitions from the implant platform through the peri‐implant mucosa toward the cervical crown form. This region corresponds to the implant supracrestal complex, which represents the soft tissue compartment coronally to the implant platform and functions as a biological seal protecting the underlying bone [2, 4, 14, 15, 16].

Within this biologically relevant compartment, prosthetic design has been increasingly recognized as a key determinant of peri‐implant tissue health. While the microbial etiology of peri‐implant diseases is well established, emerging evidence highlights the role of prosthetic geometry in modulating plaque retention and inflammatory response [17]. A recent in vivo study reported that steeper emergence angles (> 40°) may impair self‐cleansability and promote plaque accumulation in a dose‐dependent manner with increasing emergence angles [17]. Moreover, emergence angles greater than 30° and convex restoration profiles and their proximity to the crestal bone [18] were identified as risk indicators for peri‐implantitis, independent of patient‐related factors [8, 14].

Beyond plaque accumulation, the geometry of the EP may directly influence soft tissue maturation. Convex transmucosal contours may limit soft‐tissue proliferation and reduce the available space for vascularized connective tissue. In contrast, concave configurations increase the surface area of abutments, which may positively influence the barrier epithelium, thus facilitating tissue support and stability [19]. Supporting this concept, a prospective controlled trial demonstrated that implants restored with concave emergence profiles exhibited significantly lower rates of midfacial mucosal recession compared with convex designs at three years (13.3% versus 46.7%), underscoring the relevance of restorative geometry for marginal soft‐tissue stability [6, 7, 20]. These biological considerations are clinically relevant, as the preservation of an intact epithelial barrier and connective tissue seal is essential for long‐term peri‐implant stability [21, 22].

Despite growing evidence supporting biologically oriented prosthetic design, the specific influence of concave versus convex emergence profiles on midfacial mucosal stability around implant‐supported single crowns in partially edentulous patients has not yet been systematically evaluated. Previous systematic reviews have evaluated the influence of prosthetic design parameters on peri‐implant outcomes, mainly focusing on marginal bone loss, peri‐implantitis, or peri‐implant hard and soft tissue dimensions associated with different transmucosal profile configurations [23, 24, 25, 26] However, none specifically addressed midfacial mucosal stability, despite its critical role in the esthetic success of single‐tooth implant restorations. Therefore, a systematic evaluation of the available evidence is warranted to support an evidence‐based decision in clinical practice. Therefore, the aim of the present systematic review was to assess the influence of concave versus convex emergence profiles on midfacial mucosal stability in partially edentulous patients rehabilitated with implant‐supported single crowns.

## 2 Materials and Methods

The protocol of this review was registered in the International Prospective Register of Systematic Reviews (PROSPERO) with the identification code CRD420251139042 on 2 September 2025. This review adheres to the guidelines of the Preferred Reporting Items of Systematic Reviews and Meta‐Analyses (PRISMA) 2020 statement [27].

### 2.1 Eligibility Criteria and Focused Question

The following PICO framework was designed to address the review focused question:

- POPULATION (P): Partially edentulous patients rehabilitated with an implant‐supported single crown.
- INTERVENTION (I): Concave emergence profile.
- COMPARISON (C): Convex emergence profile.
- OUTCOMES (O): Midfacial mucosal stability.

In partially edentulous patients receiving implant‐supported single crowns (P), how does a concave (I), compared with a convex or non‐concave emergence profile (C), affect midfacial mucosal stability (O) in randomized controlled trials (S)?

### 2.2 Search Strategy

**FIGURE 1:**
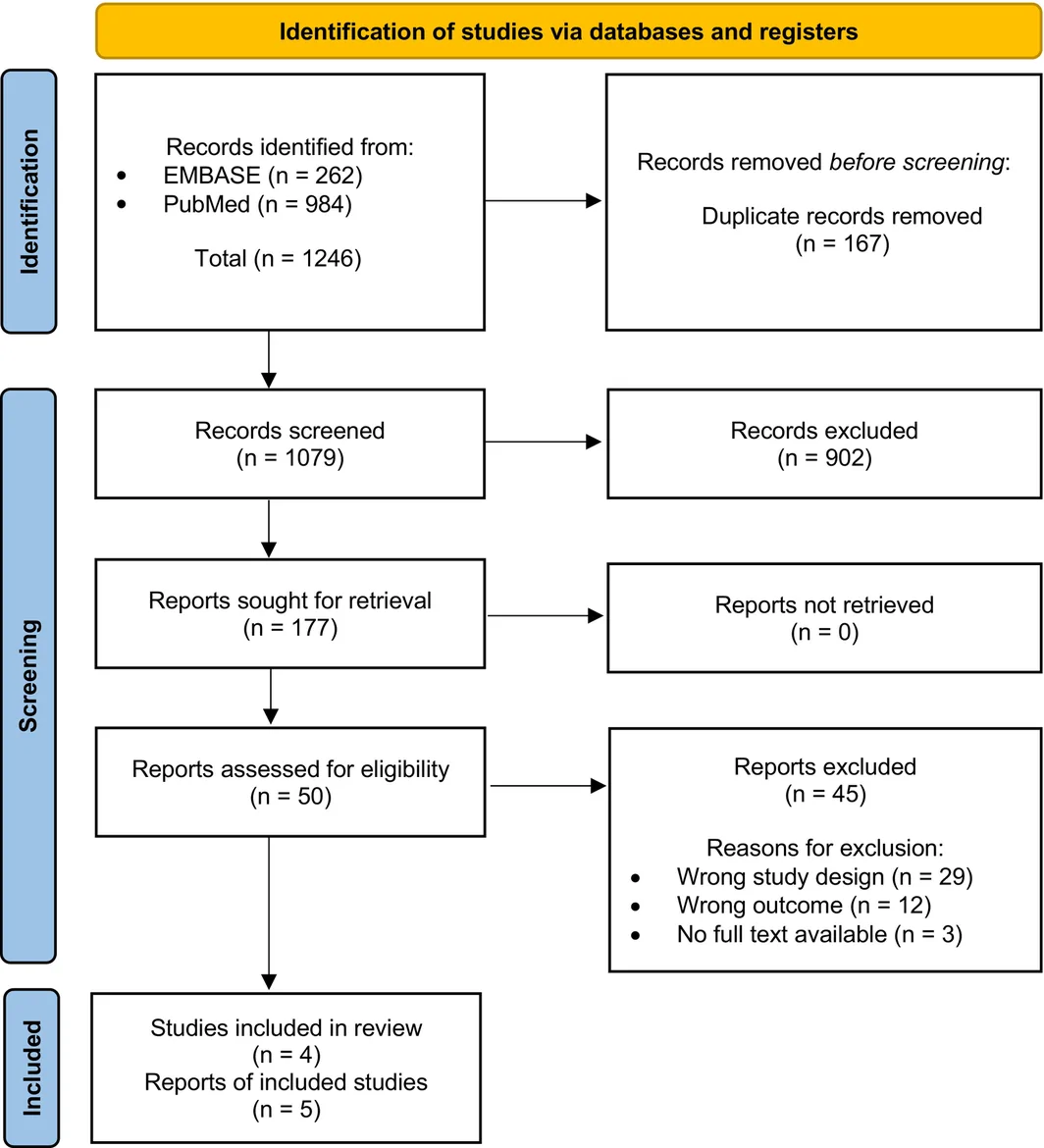
PRISMA 2020 flow diagram illustrating the study selection process. Following database searching, records were screened based on title and abstract, and full‐text articles were assessed for eligibility according to the predefined inclusion and exclusion criteria.

An electronic literature search was carried out in duplicate by two independent review authors (MP, PB) in MEDLINE (via PubMed) and Embase on February 6, 2026 and updated on May 7, 2026 (Figure 1). In addition, reference lists of retrieved studies for full‐text screening and previous reviews on the topic were screened.

The search strategy was developed in accordance with established systematic review methodology and combined Medical Subject Headings (MeSH), whereas the search strategy was adapted for Embase using Emtree terms and database‐specific field operators (e.g., ti, ab, kw) [27]. Controlled vocabulary was supplemented with free‐text terms to ensure comprehensive retrieval of relevant studies, particularly for concepts not fully represented within the indexed terminology, such as emergence profile and emergence angle.

#### 2.2.1 Search Strategy PubMed

(“dental implants”[MeSH Terms] OR “dental implant*”)

AND (abutment* OR crown* OR prosthes* OR FDP OR PFM OR bridge* OR restoration*)

*AND (emergence profile OR restorative contour OR subgingival contour OR concave OR convex OR converging OR diverging OR emergence angle OR transmucosal profile OR transmucosal design OR profile design) AND (soft tissue OR mucosal OR gingival OR phenotype OR esthetic OR esthetic OR pink esthetic score OR PES OR recession OR volume OR connective tissue OR gingival margin)*

#### 2.2.2 Search Strategy EMBASE

(‘dental implant’/exp OR ‘dental implant*’:ti,ab,kw) AND (abutment*:ti,ab,kw OR crown*:ti,ab,kw OR prosthes*:ti,ab,kw OR fdp:ti,ab,kw OR pfm:ti,ab,kw OR bridge*:ti,ab,kw OR restoration*:ti,ab,kw) AND (‘emergence profile’:ti,ab,kw OR ‘restorative contour’:ti,ab,kw OR ‘subgingival contour’:ti,ab,kw OR concave:ti,ab,kw OR convex:ti,ab,kw OR converging:ti,ab,kw OR diverging:ti,ab,kw OR ‘emergence angle’:ti,ab,kw OR ‘transmucosal profile’:ti,ab,kw OR ‘transmucosal design’:ti,ab,kw OR ‘profile design’:ti,ab,kw) AND (‘soft tissue’:ti,ab,kw OR mucosal:ti,ab,kw OR gingival:ti,ab,kw OR phenotype:ti,ab,kw OR esthetic:ti,ab,kw OR esthetic:ti,ab,kw OR ‘pink esthetic score’:ti,ab,kw OR pes:ti,ab,kw OR recession:ti,ab,kw OR volume:ti,ab,kw OR ‘connective tissue’:ti,ab,kw OR ‘gingival margin’:ti,ab,kw)

### 2.3 Inclusion and Exclusion Criteria

This systematic review included human clinical studies involving dentate adults of any age, sex and ethnicity. The types of studies considered for inclusion were randomized controlled trials (RCTs). All reviews (systematic, scoping, narrative), case reports and in vitro were excluded.

### 2.4 Study Selection

Based on the inclusion and exclusion criteria, the titles and abstracts were screened independently by two calibrated review authors (MP, PB) using the Rayyan website (https://rayyan.ai). Inter‐examiner calibration was achieved with the presence of another co‐author (FJS) by open discussion and comparison after independent assessment of the first 100 records. Any disagreement between the two reviewers was discussed and resolved by a third reviewer (AZ). Subsequently, the same authors read individually through the full‐text version of the potentially eligible studies after the calibration of five articles. When disagreement regarding the inclusion of a specific article occurred, both reviewers had an open discussion. If no agreement was achieved, another co‐author (FJS) made the final decision.

### 2.5 Data Extraction and Management

All studies meeting the inclusion criteria were included and underwent data extraction and assessment for risk of bias. Prior to commencing the data extraction process, the tables were pilot tested by two extractors. Data were independently extracted by three reviewers (MP, FJS, and AZ) and the necessary information from the included studies was imported into the data extraction table. Any disagreements were resolved through discussion.

For each study, the following data were recorded: Study design, population (number of implants, implant site, intervention, comparison, prosthetic design, follow‐up time, outcomes).

In cases of multiple publications from the same patient pool, the data was extracted from the most appropriate publication follow‐up for the meta‐analysis.

### 2.6 Risk of Bias Assessment

The assessment of methodological quality and risk of bias of the included randomized controlled trials was assessed independently by two reviewers (MP and FJS) using version 2 of the Cochrane Risk of Bias tool (RoB 2) [28]. The RoB 2 tool evaluates five domains of bias: (1) bias arising from the randomization process; (2) bias due to deviations from intended interventions; (3) bias due to missing outcome data; (4) bias in the measurement of the outcome; and (5) bias in the selection of the reported result.

Based on this assessment, each study was classified as presenting a low risk of bias when all domains were judged as low risk; some concerns when at least one domain raised concerns but none were judged as high risk; or high risk of bias when at least one domain was judged as high risk, or when multiple domains raised concerns that collectively lowered confidence in the results.

### 2.7 Statistical Analysis

To summarize and compare the studies, we pooled and analyzed the mean values of midfacial recession using weighted mean differences (MDs) with 95% confidence intervals (CIs). When studies did not provide means and standard deviations (SDs), we calculated these values by converting standard errors (SEs) or confidence intervals (CIs) as available, following the formulas in the Cochrane Handbook (section 7.7.3.2) assuming a conservative correlation coefficient of 0.5, making this result an approximate. Data were pooled using a random‐effects meta‐analysis, which adopts a conservative approach by accounting for potential variability across study settings [29, 30]. For all the meta‐analyses, heterogeneity was estimated using the restricted maximum likelihood (REML) method, recommended over DerSimonian and Laird in cases of high heterogeneity in treatment effects across studies or the number of studies is small [31]. To assess the robustness of the pooled results, we conducted a leave‐one‐out (LOO) sensitivity analysis.

To evaluate consistency across studies and quantify statistical heterogeneity, we used the *I* ^2^ statistic (Cochrane Handbook version 6.4, 2023), with the following interpretation thresholds for heterogeneity: 0%–40% (low), 30%–60% (moderate), 50%–90% (possible substantial), and 75%–100% (considerable heterogeneity). All meta‐analyses were carried out using Stata 19.5 software (StataCorp, College Station, TX).

## 3 Results

### 3.1 Study Selection

From the initial 1246 records identified, 167 duplicates were removed, resulting in 1079 records for title screening. Of these, 1079 records were excluded as they did not meet the inclusion criteria. Subsequently, 177 abstracts were assessed, leading to the exclusion of an additional 127 records. A total of 50 full‐text articles were retrieved and evaluated for eligibility, of which 5 reports representing 4 independent studies were included in the final analysis. The study selection process is illustrated in Figure 1.

### 3.2 Study Characteristics

Table 1 summarizes the general details, subjects' information and prosthetic design of the included studies. The four included RCTs evaluated the influence of transmucosal prosthetic design (abutments) on peri‐implant soft tissue outcomes in partially edentulous patients rehabilitated with implant‐supported single restorations. Across all the studies, a total of 144 implants were placed and restored following surgical and prosthetic protocols, with a follow‐up period of 12 months. Distribution of implants included in the quantitative synthesis according to emergence profile group is summarized in Table S1.

**TABLE 1:** Characteristics of the included randomized controlled trials (RCT) (*n* = 4).

Study	Study design	Population (n/implants)	Implant site	Intervention	Comparison	Prosthetic design	Follow‐up	Outcomes
Barwacz et al., 2025	RCT	54 patients/54 implants	Partially edentulous anterior maxilla (first premolar to first premlolar)	CAD/CAM abutment (concave morphology)	Standardized abutment design (linear divergent morphology)	Zirconia abutment with concave or linear diveregent morphology influencing emergence profile	12 months	Soft tissue stability, marginal bone levels (indications for peri‐implant health)
Koutouzis et al., 2019	RCT	26 patients/26 implants	Partially edentulous maxillary premolar region	Abumtent with convex emergence shape	Abumtent with concave emergence shape	Abutment contour influencing peri‐implant tissue level	12 months	Clinical and radiographic peri‐implant tissue changes
Siegenthaler et al., 2022	RCT	44 patients/44 implants	Anterior area of mandible and maxilla (first premolar to first premlolar)	Convex emergence profile	Concave emergence profile	Emergence provile influences mucosal margin stability	12 months	Clinical, esthetic and profilometric outcomes, presences of recessions
Wang et al., 2022	RCT	19 patients/20 implants	Partially edentulous mandiular molar region	Buccal convex profile	Buccal originale emergence profile	Emergence profile design	12 months	Ginigival margin level change, marginal bone loss, implant failure, complications

*Note:* The table summarizes the study design, study population, implant site, intervention and comparison, prosthetic design, follow‐up period, and reported outcomes for each included study (*n* = 4).

Abbreviations: CAD/CAM, computer‐aided design/computer‐aided manufacturing; EP, emergence profile; MBL, marginal bone level; mm, millimeters; n, number of patients; RCT, randomized controlled trial.

The interventions assessed comprised different emergence profile configurations and abutment macro‐designs, representing either concave or convex transmucosal contours, or morphologies expected to influence the prosthetic profile at the peri‐implant mucosal interface. While two trials directly compared concave versus convex emergence profiles [6, 32], the remaining studies assessed CAD/CAM or prefabricated abutment designs with distinct contour characteristics [33, 34], thereby indirectly modifying the emergence profile.

All included studies were conducted in controlled clinical settings and involved implant placement in healed or post‐extraction sites, predominantly in esthetically relevant regions. Restorations consisted of single implant‐supported crowns and standardized clinical protocols were applied for prosthetic delivery.

The primary outcomes assessed across studies included midfacial mucosal level changes, peri‐implant soft tissue dimensions and in some cases, radiographic marginal bone level changes. Soft tissue changes were reported in millimeters and were evaluated using clinical measurements and/or standardized photographic analysis.

Despite minor heterogeneity in intervention definitions and outcome assessment methods, all studies assessed the role of prosthetic contouring at the transmucosal level as a determinant of peri‐implant soft tissue stability (midfacial mucosal recessions), allowing for quantitative comparison and meta‐analysis.

### 3.3 Risk of Bias Assessment

**FIGURE 2:**
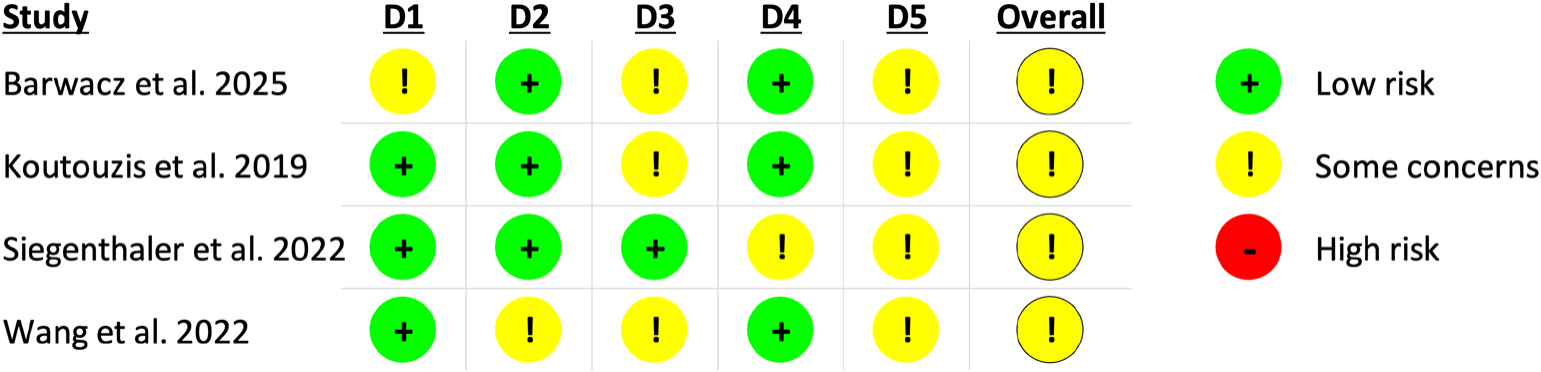
Risk of bias assessment of the included randomized controlled trials using the Cochrane Risk of Bias 2 (RoB 2) tool. Each domain was evaluated as low risk (green), some concerns (yellow), or high risk of bias (red). The domains assessed included bias arising from the randomization process (D1), deviations from intended interventions (D2), missing outcome data (D3), measurement of the outcome (D4), and selection of the reported result (D5). Overall, the included studies were judged to present mainly some concerns, with variability across individual domains.

The risk of bias assessment of the included studies revealed that all RCTs were judged to present some concerns. None of the studies fulfilled the criteria for a low risk of bias across all domains and no study was classified as having a high risk of bias (Figure 2).

Across the individual domains of the RoB 2 tool, the overall judgment was primarily influenced by insufficient reporting of allocation concealment procedures, the presence of missing outcome data and limitations related to outcome measurement and statistical analysis. In particular, concerns frequently arose in the domains of bias due to missing outcome data and bias in the selection of the reported result, often related to the absence of a clearly reported pre‐specified analysis plan or protocol (Figure 2).

### 3.4 Meta‐Analysis

**FIGURE 3:**
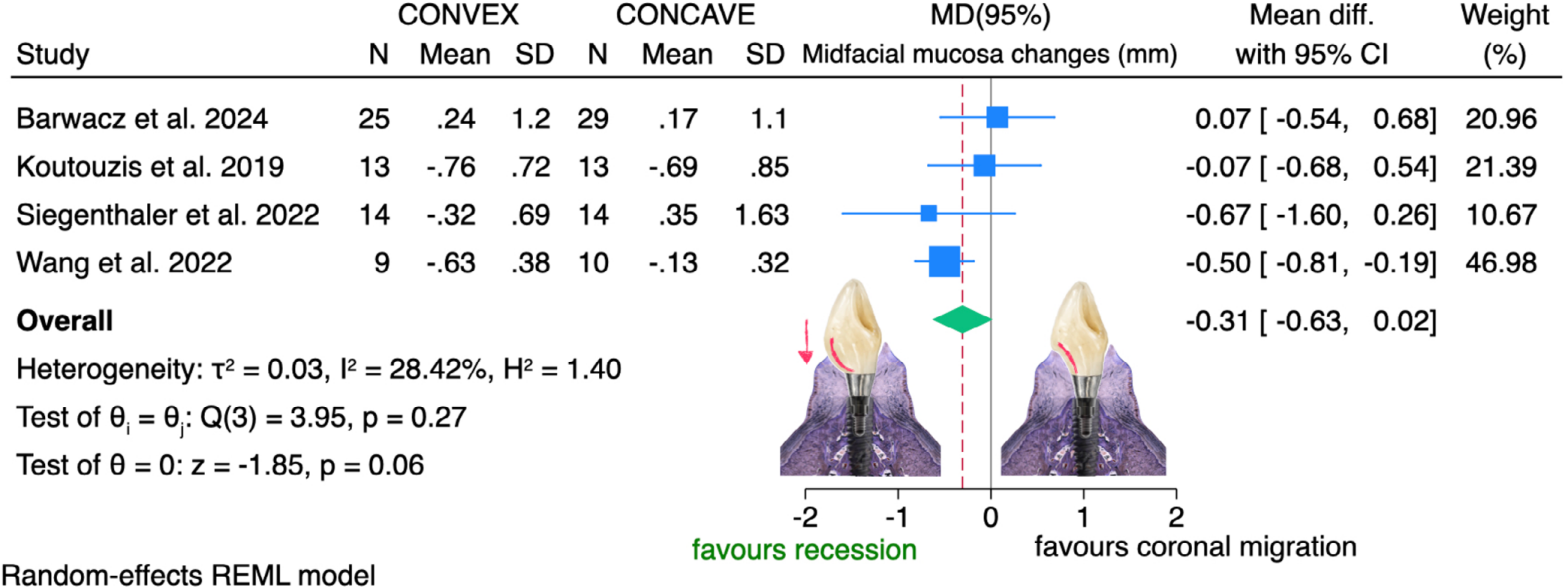
Forest‐plot for midfacial mucosal changes in the CONVEX and CONCAVE groups after 12 months. Boxes and pooled estimate (diamond) calculated by the random effects restricted maximum likelihood approach. Horizontal bars and diamond widths denote 95% CIs, and box sizes indicate relative weight in the analysis. MD: Mean difference; SD: Standard deviation.

A random‐effects meta‐analysis including four studies assessed changes in marginal soft‐tissue levels measured in millimeters (mm). The pooled analysis showed a mean difference of −0.31 mm (95% confidence interval −0.63 to 0.02) when comparing convex emergence profiles with concave profiles (Figure 3). This negative pooled estimate indicates a trend toward greater peri‐implant soft‐tissue recession with convex profiles, with borderline statistical significance (*p* = 0.064) (Figure 3).

Between‐study heterogeneity was low (*I* ^2^ = 28%) and the test for heterogeneity was not significant (*Q* = 3.95; *p* = 0.27), suggesting broadly consistent findings across studies. Overall, the results indicate a clear directional trend toward midfacial recession (≈0.3 mm) when convex emergence profiles are used compared with concave profiles.

### 3.5 Sensitivity Analysis

**FIGURE 4:**
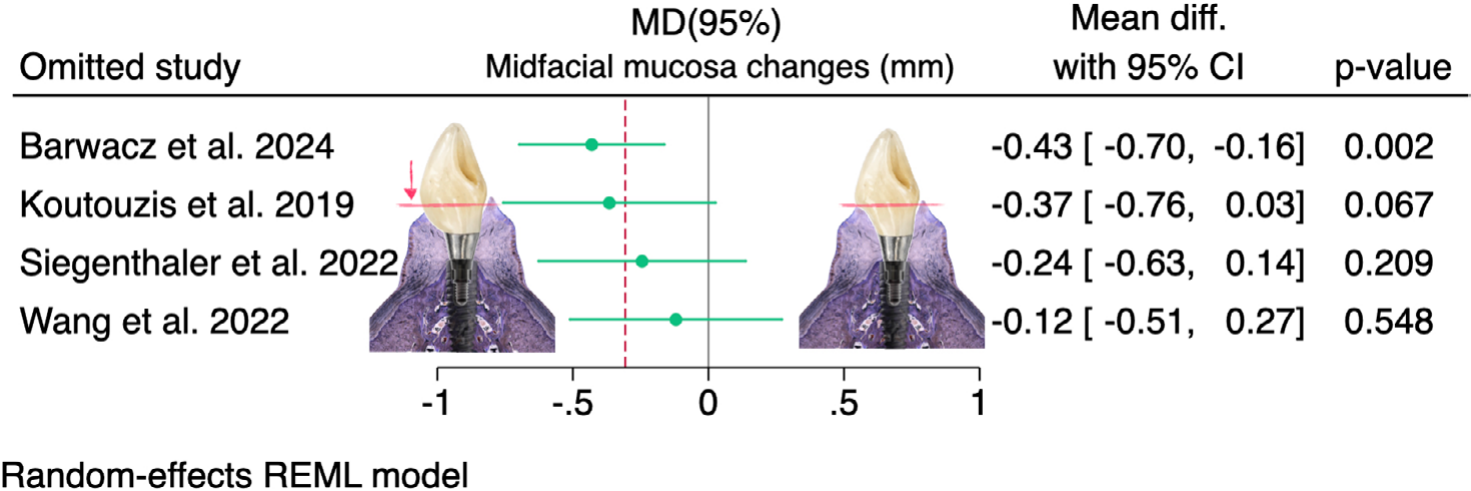
Leave‐one‐out sensitivity analysis of the meta‐analysis evaluating the effect of emergence profile on midfacial mucosal changes.

To estimate the accuracy and robustness of the estimated treatment effect, we excluded studies from the meta‐analysis one by one by applying the leave‐one‐out analysis. A leave‐one‐out sensitivity analysis was performed to assess the influence of individual studies on the pooled estimate. Sequential omission of each study produced pooled mean differences ranging from −0.12 mm to −0.43 mm, with the direction of effect consistently favoring greater midfacial recession with convex emergence profiles compared with concave profiles. Exclusion of Barwacz et al. [33] strengthened the association and resulted in a statistically significant pooled estimate (−0.43 mm; 95% CI −0.70 to −0.16; *p* = 0.002), whereas omission of Koutouzis et al. [32] or Siegenthaler et al. [6] produced similar but less precise estimates that did not reach statistical significance (Figure 4).

When Wang et al. [34], the study contributing the largest weight in the primary analysis, was excluded, the pooled estimate was attenuated (−0.12 mm; 95% CI −0.51 to 0.27; *p* = 0.548), although the direction of effect remained unchanged. Overall, these analyses indicate that the direction of the pooled effect was consistent across iterations, with estimates generally favoring greater recession with convex profiles.

## 4 Discussion

This systematic review and meta‐analysis evaluated the influence of concave versus convex emergence profiles on midfacial mucosal stability in partially edentulous patients rehabilitated with implant‐supported single crowns. Overall, the findings suggest that convex emergence profiles are associated with a greater tendency toward midfacial mucosal recession than concave profiles in the short term.

Previous systematic reviews have mainly focused on emergence angle, contour, or restorative design in relation to peri‐implant bone loss and peri‐implantitis, consistently suggesting that overcontoured or convex transmucosal profiles may be associated with increased biological complications [23, 24, 25].

However, none specifically addressed midfacial mucosal stability, despite its critical importance for the esthetic success of single implant‐supported restorations. To the best of our knowledge, this is the first meta‐analysis specifically assessing the influence of emergence profile design on peri‐implant soft tissue stability, showing a consistent tendency toward greater midfacial recession in association with convex or nonconcave emergence profiles. Together, these findings support the concept that prosthetic contouring may influence not only plaque accumulation and inflammation [17], but also the adaptive capacity and stability of the peri‐implant soft tissues.

From a clinical perspective, these results suggest that emergence profile design is not merely a prosthetic consideration but may play a role in maintaining peri‐implant soft tissue stability. Although the magnitude of the observed differences was modest, the direction of the effect was consistent across sensitivity analyses, supporting the robustness of the findings. Consequently, concave emergence profiles may be preferred to minimize the risk of midfacial soft tissue recession as indicated by previous anecdotal evidence [1, 2, 3, 4, 15, 16, 35, 36, 37].

These findings should be interpreted within the broader context of peri‐implant tissue stability, which is influenced by multiple biological and prosthetic factors [5]. These include three‐dimensional implant positioning, the quantity and quality of hard and soft tissues and prosthetic design characteristics such as abutment morphology, emergence angle, abutment height, material selection and handling. From a clinical perspective, the three‐dimensional position of the implant is among the most critical determinants, as it directly affects prosthetic design and many of the factors involved in maintaining peri‐implant tissue stability [38, 39]. Improper implant positioning may compromise the space required for adequate tissue dimensions, leading to shimmering of the abutment, gray discoloration of the peri‐implant tissues, midfacial recession, facial dehiscence and an unfavorable peri‐implant environment [40].

Several biological and prosthetic factors may explain the tendency toward greater midfacial mucosal recession observed with convex emergence profiles. Prosthetic contouring at the transmucosal level has been shown to influence both the stability of the peri‐implant mucosa [2, 36, 41] and its dimensions [42, 43]. One possible explanation is that convex emergence profiles reduce the available space for soft‐tissue maturation during healing. By occupying more of the peri‐implant soft tissue envelope, convex contours may limit the capacity for coronal tissue adaptation and the phenomenon often described as “soft‐tissue creeping” [44]. In contrast, concave emergence profiles may provide additional space, or “running room,” for soft‐tissue accommodation, facilitating coronal tissue adaptation and potentially promoting more stable midfacial mucosal levels over time [44, 45]. Early clinical observations support this concept, with relatively low rates of recession reported around implants restored with concave emergence profiles [20].

Experimental evidence further supports this biological rationale. Preclinical studies suggest that the restorative angle at the transmucosal level may influence the organization of the peri‐implant soft tissue interface. Narrower restorative angles, which are characteristic of concave profiles, appear to favor the formation of a structured peri‐implant soft tissue barrier resembling the classical cuff‐like attachment around implants [19, 22, 46, 47, 48]. In contrast, wider restorative angles, more representative of convex profiles, have been associated with disruption of the junctional epithelium's continuity and reduced integrity of the peri‐implant soft tissue seal [22]. Such alterations may compromise the peri‐implant mucosa's ability to maintain a stable marginal position [49], thereby increasing the likelihood of recession.

All but one study [33] demonstrated a clear advantage of concave emergence profiles in maintaining midfacial mucosal stability. The lack of significant differences reported in the remaining study is most likely related to the minimal morphological contrast between the evaluated interventions, which compared a concave profile with a straight (linearly divergent), rather than a convex, design. In the present review, convex and concave emergence profiles were compared, representing two distinct and opposing transmucosal geometries. In contrast, the RCT by Barwacz et al. compared concave and linearly divergent profiles, which are relatively similar in shape and may therefore exert comparable effects on peri‐implant soft tissues. Consequently, the limited geometric contrast between the interventions in that study may have reduced its ability to detect clinically meaningful differences.

From a clinical standpoint, these findings highlight the importance of integrating surgical and prosthetic planning. Implant positioning, abutment selection and emergence profile design should not be considered in isolation, but rather as interdependent components of a biologically and esthetically driven treatment concept. In particular, the use of concave emergence profiles during provisionalization and in the definitive restoration may support midfacial mucosal stability, especially in esthetically demanding regions. At the same time, the implant‐supported restoration and the surrounding peri‐implant tissues should be understood as a framework in which several parameters interact to determine the final outcome. These include, but are not limited to, the three‐dimensional implant position, the biological characteristics of the peri‐implant tissues in terms of quality and quantity and the prosthetic design of the suprastructure. Only when these factors are carefully coordinated, while respecting biological principles and following “common sense”, can they be maintained in a state of harmony, ultimately enabling predictable long‐term stability and favorable esthetic outcomes.

This systematic review and meta‐analysis has several limitations, including the limited number of available RCTs, relatively small sample sizes and short follow‐up. In addition, emergence profile designs were not standardized across studies, which may partly explain the limited detrimental effect observed with linearly divergent or nonconcave profiles. While some studies directly compared clearly distinct concave and convex contours, others evaluated modified or biologically guided transmucosal designs with only minor anatomical differences between groups. Nevertheless, the consistent direction of effect across most studies supports the presence of a true biological association. Additional heterogeneity may have arisen from differences in prosthetic components, including abutment design, abutment height and implant systems, as well as from variations in outcome assessment methods, such as digital model analyses and clinical measurements. Finally, a number of included studies presented some concerns regarding risk of bias. All these limitations should be considered when interpreting the pooled estimates.

## 5 Conclusions

Convex emergence profiles around dental implants appear to be associated with a tendency toward midfacial mucosal recession compared with concave profiles in the short term.

## Funding

The authors have nothing to report.

## Disclosure

The authors do not have any financial interest in the companies whose materials are included in this article.

## Conflicts of Interest

The authors declare no conflicts of interest.

## Supporting information


**Table S1:** Distribution of implants included in the quantitative synthesis according to emergence profile group. The table presents the number of implants allocated to the control (convex/non‐concave) and test (concave/modified) groups for each study included in the meta‐analysis.

## Data Availability

The data that support the findings of this study are available on request from the corresponding author. The data are not publicly available due to privacy or ethical restrictions.
