# Supplementary material for: Influence of Concave Versus Convex Emergence Profiles on Midfacial Mucosal Stability—A Systematic Review With Meta‐Analysis
Source: J Esthet Restor Dent. 2026 Jun 20;38(10):1910–9. doi: 10.1111/jerd.70210 (PMC13576815; doi:10.1111/jerd.70210)
Supplement: Supplementary file 1 [file JERD-38-1910-s001.docx]

**Supplementary Table S1.** Distribution of implants included in the quantitative synthesis according to emergence profile group. The table presents the number of implants allocated to the control (convex/non-concave) and test (concave/modified) groups for each study included in the meta-analysis.

| **Study** | **Control / convex or non-concave** | **Test / concave or modified profile** |
| --- | --- | --- |
| Barwacz et al. 2025 | 25 implants | 29 implants |
| Koutouzis et al. 2019 | 13 implants | 13 implants |
| Siegenthaler et al. 2022 | 14 implants | 14 implants |
| Wang et al. 2022 | 10 implants | 10 implants |
